# The Effect of Early Life Factors and Early Interventions on Childhood Overweight and Obesity

**DOI:** 10.1155/2015/964540

**Published:** 2015-03-25

**Authors:** Li Ming Wen, Chris Rissel, Gengsheng He

**Affiliations:** ^1^Department of Endocrinology & Metabolism, Shanghai 10th People's Hospital, University of Tongji, Shanghai 200072, China; ^2^Sydney Medical School, University of Sydney, Sydney 2006, Australia; ^3^Health Promotion Service, Sydney Local Health Districts, NSW 2050, Australia; ^4^Prevention Research Collaboration, Sydney School of Public Health, The University of Sydney, Sydney 2006, Australia; ^5^School of Public Health, Fudan University, Shanghai 200032, China

The global obesity epidemic continues to be one of the major health burdens on society, which directly affects our younger generation. Worldwide, more than 40 million children under the age of five were overweight with a global prevalence of overweight being 6.7% in 2010 [1]. The rates of overweight and obesity have been dramatically increasing in some developing countries such as China [2] over recent years. This highlights the need for a better understanding of the effect of early life factors on overweight and obesity and, more importantly, developing effective early interventions. 

Research evidence has already showed that some early infant feeding practices, including breastfeeding and the timing of the introduction of solids as well as children's eating habits and television viewing time, are among the most identifiable factors contributing to early onset of childhood obesity [3]. However, these risk factors for overweight and obesity are just the tip of the iceberg. There remain many unknown factors which require further research. In particular, the context of early life factors includes family environments and parental inferences as well as societal influences. With this in mind we selected the theme for this special issue.

There has been an excellent response, with various research ideas and approaches and the authors' making important contributions to this special issue. We wish to highly commend the authors for their well written papers exploring a range of issues related to early life factors associated with childhood obesity, which include epidemiological investigation, laboratory research, and intervention studies at various life-stages such as gestation period, infants, childcare, primary, and adolescents.

J. A. W. Baidal et al. examined underlying reasons for early life obesity risk factors and identify potential early life intervention strategies by conducting 7 focus groups with 49 Hispanic women who were pregnant or had children of age < 24 months. They concluded opportunities exist in the first 1000 days to improve Hispanic mothers' understanding of the role of early life weight gain in childhood obesity and other obesity risk factors. K. N. Dancause et al. assessed degree of objective hardship and subjective distress in women pregnant during severe flooding. Their results support the hypothesis that prenatal stress increases adiposity beginning in childhood and suggest that early gestation is a sensitive period.

Adolescence is a much neglected focus for overweight and obesity research. K. J. Sawka et al. address this gap by using social network analysis to examine how friendships affect sedentary behaviour and physical activity levels. Examining a cross-sectional analysis of 1061 adolescents (11–15 years), they found that adolescents with no friendship nominations participated in less moderate-vigorous physical activity (MVPA). A. L. Rodríguez-Ventura et al. also explored the barriers to losing weight experienced by children and adolescents in Mexico from the perspective of the children/adolescents and their parents using a series of focus groups. They found that barriers to losing weight included a perception that they were not overweight and not recognising overweight or obesity as a disease with serious consequences.

Very early overweight prevention programs can begin during pregnancy, with a focus on healthy weight gain by the mother. E. Y. Lau et al. conducted a systematic review of the literature on the association between gestational weight gain (GWG) and offspring's body weight with a focus on prospective and retrospective cohort studies. The authors found that total GWG and exceeding the recommendation were associated with higher BMI *z*-score and elevated risk of overweight or obesity in offspring.

Acknowledging that parents play a critical role in developing and shaping their children's physical activity and sedentary behaviours, particularly in the early years of life, H. Xu et al. also conducted a systematic review of associations of parental influences with physical activity and screen time among young children. Their findings suggest that parents' encouragement and support can increase children's physical activity, and reducing parents' own screen time can lead to decreased child screen time.

A. D. Keita et al. report on the feasibility and acceptability of a new early intervention program, the Healthy Homes, Healthy Families Pilot Study, which builds on the success of the home-based Healthy Beginnings program [4]. The Healthy Homes, Healthy Families Pilot Study, was designed to empower low-income racially/ethnically diverse parents to modify their children's health behaviours. They found vegetable intake among children significantly increased at follow-up, and fruit juice consumption decreased. J. Bonnet et al. also focused on early intervention and the idea of introducing discussion with parents of children's healthy weight when the child is 12 months old. They identified that children at this age were already demonstrating poor nutrition and physical activity behaviours and proposed that talking with parents at the 12-month visit with the family medicine primary care providers about “French fries” and nutrition was potentially an important opportunity for health promotion.

Using theory is important in developing and evaluating programs, and R. Lakshman et al. used the Medical Research Council framework for the development and evaluation of complex interventions: the baby milk intervention and trial. They reviewed the epidemiological evidence on early life risk factors for obesity and interventions to prevent obesity in this age group and identified the prevention of excess weight gain in bottle-fed babies and appropriate weaning as intervention targets. They developed intervention materials and evaluation tools and conducted qualitative studies with mothers (intervention recipients) and healthcare professionals (intervention deliverers) to refine them. Evaluation will follow, but it was noted that the rigorous development process was resource intensive.

A. C. Lindsay et al. described how childcare providers play an influential role in promoting healthful eating and physical activity behaviors of preschool children in their care. They also identified many barriers and challenges in establishing and maintaining healthful nutrition and physical activity behaviors, including high cost of healthy foods, cold weather, and physical environment of childcare centers and homes. K. Röttger et al. also sought to explore physical activity in the preschool setting, as this can be a time when gross motor skills are developed. Using direct accelerometry and anthropometrical and family-related data, they compared physical activity levels of 114 children attending different preschool settings in four cities of the trinational Upper Rhine region (Freiburg, Landau/Germany, Basel/Switzerland, and Strasbourg/France). Children from Strasbourg and Landau were significantly more passive than children from Basel and Freiburg, with the authors concluding that more open preschool systems such as those in Basel, Freiburg, and Landau did not lead to more physical activity “per se” compared to the highly regimented desk-based system in France.

School based programs continue to be important, and S. Kobel et al. report on the results of primary school focused program “Join the Healthy Boat.” This teacher delivered program focused on increasing physical activity, decreasing screen media use, more regular breakfast, and reducing the consumption of soft drinks. 1943 primary school children participated in the cluster-randomised study. Significant effects were found in the intervention group for screen media use among girls, for nonmigrant children, and children with parents having a low education level.

While recognising overweight and obesity as significant public health problems, S. N. Saeidlou et al. sought to examine the more traditional developing country problem of malnutrition. Working with the Office of Community Nutrition Improvement and the United Nations Children's Fund, they conducted a prevalence study of malnutrition and obesity in 902 children under 5 years old in the Salmas district of North West Azerbaijan, Iran, in 2011. The prevalence of obesity and overweight in children was 1.3% and 5.1%, respectively. They found that the prevalence of malnutrition based on underweight, stunting, and wasting was estimated to be 2.3%, 7.3%, and 1.4% among children, respectively, and was more common in rural areas.

*Li Ming Wen* *Li Ming Wen* *Chris Rissel* *Chris Rissel* *Gengsheng He* *Gengsheng He*
